# How Should Self-Esteem Be Considered in Cancer Patients?

**DOI:** 10.3389/fpsyg.2021.763900

**Published:** 2021-10-28

**Authors:** Noémie Niveau, Boris New, Marine Beaudoin

**Affiliations:** ^1^LPNC, CNRS, Univ. Savoie Mont-Blanc, Chambéry, France; ^2^LIP/PC2S, Univ. Savoie Mont-Blanc, Chambéry, France

**Keywords:** cancer, self-esteem, depression, coping, social support

## Abstract

Numerous studies showed that cancer significantly increases the risk of developing depressive and anxious symptoms. It has been shown that self-esteem is an important psychological resource and is associated with many health behaviors. Furthermore, the vulnerability model of low self-esteem, which has received strong empirical support, highlights that low self-esteem is a real risk factor in the development of depressive disorders. This article aims at providing an overview of the involvement of self-esteem in the psychological adjustment to cancer. After briefly reviewing the literature, we suggest that its implication in the development of depressive disorders and its association with coping strategies and social support in cancer patients justify the consideration of self-esteem in oncology psychological care, especially in young adult patients and those with significant physical impairment following treatment.

## Introduction

The number of people affected by cancer has risen sharply in recent decades. Worldwide, it was estimated in 2018 that one in five men and one in six women will develop cancer in their lifetime (Bray et al., 2018). According to the International Agency for Research on Cancer, in 2020, there were more than 19 million new cases of cancer in the world (Ferlay et al., 2020). Psychological care is frequently offered to oncology patients because of the significant psychological impact of cancer diagnosis and treatment. For example, studies have shown a decrease in quality of life (Kwan et al., 2010; Leung et al., 2014) and well-being for up to 4years after diagnosis (Janz et al., 2014) in patients with breast cancer.

Many studies have shown the role of self-esteem in the development of psychological disorders (Sowislo and Orth, 2013). By reviewing the contributions of published articles on self-esteem and psychological adjustment in cancer patients, we sought to establish if cancer could impact self-esteem and, if so, whether this could explain the important prevalence of depressive disorders in patients. Considering its frequent association with the patients’ psychological adjustment strategies and social support, we argue that it remains necessary to consider self-esteem in some at-risk patients.

## Psychological Adjustment to Cancer

Depressive disorders are considered a frequent consequence of cancer diagnosis and treatment (Pasquini and Biondi, 2007). Comparing cancer patients with “healthy” subjects (no diagnosed physical or psychiatric pathology), different studies have shown a significantly higher depressive symptom score in cancer patients at 3months after diagnosis (Schroevers et al., 2003a,b, 2006). Lo et al. (2010) also showed important rates of depressive symptoms in patients with severe cancer (lung cancer and metastatic gastrointestinal cancer). A longitudinal study showed that while anxiety symptoms tend to decrease 6months after the end of treatment, the level of depression in breast cancer patients remains high long after treatment has ended (Moreira and Canavarro, 2010). These various studies highlight the heavy impact of cancer on patients’ mental health.

Coping is generally defined as the efforts made to deal with threatening or harmful situations to reduce the negative psychological impact (Carver and Vargas, 2011; Kreitler, 2019). Coping strategies have a significant positive effect on the level of psychological distress, depression, anxiety, and quality of life of cancer patients (Fasano et al., 2020), as well as on the patients’ health status (Kvillemo and Bränström, 2014). Coping strategies can take many forms and vary according to people and situations (Lazarus and Folkman, 1984; Jenkins and Pargament, 1988). For example, it has been shown that the patient’s perception of his or her cancer (threat, loss, and challenge) determine the type of coping strategy that he or she implement (Franks and Roesch, 2006). However, some coping strategies may generally be more effective in reducing psychological distress in patients. A meta-analysis showed that approach-based coping strategies are more effective on the psychological adjustment of prostate cancer patients than avoidance-based coping (Roesch et al., 2005). In the case of breast cancer, coping strategies based on emotion (e.g., emotional regulation, relaxation) seems particularly adapted to reduce psychological distress by focusing not on the stressor as such (i.e., cancer) but on the induced emotions. It also has been shown that acceptance of the disease, positive reappraisal, and use of humor have positive effects on psychological distress in breast cancer patients (Carver et al., 1993).

Social support is another coping strategy (Thoits, 1986) that helps people defend against a stressful event, such as cancer (Wilcox, 1981; Cohen and Wills, 1985; Hobfoll et al., 1990). A multitude of studies has looked at the impact of social support on the psychological condition of oncology patients. It has been shown that social support is strongly associated with patients’ quality of life (Luszczynska et al., 2013; Leung et al., 2014; Zhang et al., 2017), depressive symptoms (Hann et al., 2002), and anxiety (Hipkins et al., 2004). Importantly, social support is thought to be involved in therapeutic adherence to medical treatments (DiMatteo, 2004), which subsequently impacts disease progression (Nausheen et al., 2009; Usta, 2012).

However, coping can also have a deleterious effect, particularly when the patient wants to but is unable to implement coping strategies or uses strategies that are inappropriate for the situation. For example, denial and avoidance predict poor psychological adjustment (Dunkel-Schetter et al., 1992; Carver et al., 1993). It has also been shown that the implementation of maladaptive adjustment strategies at the time of diagnosis of the disease determines the level of depression of patients up to 1year later (Grassi et al., 1997).

## Self-Esteem as a Psychological Resource in Cancer

Self-esteem is an indicator of tolerance, acceptance, and personal satisfaction with oneself (Rosenberg, 1965) and results from the affective evaluation of the set of attributes that an individual assigns to himself. Self-esteem has been conceptualized in many theoretical approaches. Depending on these approaches, self-esteem results from the perceptions of oneself in various domains of the life and the importance, we grant to these domains (additive approach; Harter, 2006), the ideals which are associated with it (intrapersonal approach; Higgins, 1987) or the way in which we think that the others perceive us (interpersonal approach; Leary and Baumeister, 2000). Self-esteem is central to human well-being and the level of self-esteem has many consequences. For example, correlational studies showed that a high level of self-esteem leads to a sense of initiative (Baumeister et al., 2003), perseverance in the face of difficulties, an increase in efforts to achieve a goal (Di Paula and Campbell, 2002), and many health behaviors (Stinson and Fisher, 2021). Conversely, low self-esteem is associated with high frequency and intensity of physical symptoms (Robinson et al., 2006) and is a transdiagnostic symptom to many mental health disorders (Zeigler-Hill, 2011). Importantly, strong empirical support has been provided for the vulnerability model that posits that low self-esteem is a risk factor for depression (Orth et al., 2008; Sowislo and Orth, 2013; Rieger et al., 2016). People with low self-esteem seem to be more sensitive to the psychological impact of negative life events. Stake et al. (1995) identified that when faced with a negative event, participants with low self-esteem experience more negative effects than participants with high self-esteem. Furthermore, it has been shown that negative events will have a greater impact on all self-evaluations (even those that are not relevant to the situation or unrelated to the event) in subjects with low self-esteem than in those with high self-esteem (Stake et al., 1995; Greenier et al., 1999). Thus, the level of self-esteem could be a predictor of the psychological impact of life events. The Terror Management Theory considers self-esteem as a buffer against existential death anxiety (Greenberg et al., 1986; Solomon et al., 1991; Pyszczynski et al., 2004). Self-esteem is seen here as a psychological resource that would allow individuals to manage the fear caused by the awareness of an inescapable death by feeling accepted and valued in society. From this point of view, the preservation of a high level of self-esteem in patients could allow them to cope better with cancer, a pathology that can increase the salience of this death anxiety. However, findings about the consequences of self-esteem must be viewed with caution because of many limitations associated with self-esteem studies (i.e., subjective measures, confounding effects with other variables highly correlated with self-esteem; Baumeister et al., 2003).

Moreover, different studies have highlighted the association between the level of self-esteem and coping strategies in cancer patients. Cieślak and Golusiński (2018) showed that greater acceptance of cancer-related disability was associated with high self-esteem. Mingorance et al. (2019) also identified that adaptive adjustment strategies (positive reframing, use of emotional support, active coping, acceptance, and planning) in breast cancer patients were associated with high self-esteem. Social support also appears to be strongly related to self-esteem, in line with the sociometer theory, which postulates that self-esteem is an indicator of social inclusion (Leary and Baumeister, 2000). The relationship between social support and self-esteem has been widely studied in cancer patients (Curbow and Somerfield, 1991; Schroevers et al., 2003b; Mingorance et al., 2019; Pardede et al., 2020), and appears to be of prime importance. In particular, it has been shown that positive relationships with others are more strongly correlated with the level of self-esteem in female cancer patients than in age-matched healthy women (Carpenter, 1998).

The associations between self-esteem, coping, and social support allow us to question the role of this psychological resource in the development of depressive disorders in cancer patients. Various psychological processes explaining the effect of low self-esteem on the development of depression have been proposed. Some studies showed that people with low self-esteem may tend to ruminate on negative aspects of self-concept, which will lead to the development of depression (Kuster et al., 2012; Phillips and Hine, 2016). Other explanations are that people with low self-esteem may excessively seek reassurance from their loved ones (Potthoff et al., 1995) or tend to seek negative social cues from their partners to maintain consistency with their negative self-concept (Giesler et al., 1996). These may lead to a disruption of social relationships, a decrease in social support, and an increase in the likelihood of developing depression. Thus, in cancer patients, low self-esteem may contribute to decreased social support. Given the importance of social support in cancer patients, this may impair patients’ psychological adjustment to cancer, making them more vulnerable to developing depressive disorders. From this point of view, the preservation of high self-esteem in cancer patients would reduce the risk of depression associated with the illness.

## Is Self-Esteem Impaired in Cancer Patients?

It seems intuitive to think that cancer patients experience a decrease in their self-esteem given the major negative changes that the pathology involves. Social self-perceptions can be disrupted at the time of diagnosis by the coating of a “sick” status that can modify interpersonal relationships, and lead to a feeling of social isolation. Body image and physical self-perceptions can also be affected by cancer, mainly at the time of treatment, which can cause significant side effects: scars, alopecia, burns, sexual problems, pain, etc. Finally, self-efficacy can also be impacted by cancer during treatment with the feeling of significant fatigue and possible handicaps that can limit the activities of daily living.

In this line, Curbow and Somerfield (1991) postulate in their review that cancer and its treatment are important stressors that make patients particularly vulnerable to changes in self-perception. This postulate is consistent with some work that has shown the impact of negative and/or stressful life events on self-esteem (Nezlek and Plesko, 2001). Works of Curbow and Somerfield (1991) were often wrongly cited in the literature as references for the serious impact of cancer on self-esteem (Carpenter, 1998; e.g., Berterö, 2002; Lee et al., 2006; Özdemiroglu et al., 2017). However, these authors finally conclude that cancer patients do not seem to differ from healthy subjects on self-esteem. These authors also highlight that young patients are at the greatest risk of having a decrease in their level of self-esteem and that social support seems to be particularly related to patients’ self-esteem. Moreover, some studies support the findings of Curbow and Somerfield (1991) by demonstrating the preservation of self-esteem in cancer patients. For example, a cross-sectional study had shown that the level of self-esteem remains relatively high in breast cancer patients undergoing treatment (Shafaee et al., 2018). Another study has shown that the level of self-esteem of breast cancer patients was slightly higher than that of age-matched healthy subjects (Carpenter, 1998). Using a longitudinal design, a cross-sectional study incorporating all types of cancer had also identified that patients’ level of self-esteem at 3months, 15months, and 8years after diagnosis was not significantly different from age and gender-matched healthy subjects (Schroevers et al., 2003b, 2006). However, considering the multiple factors involved in self-perceptions, the impact of cancer on self-esteem can be modulated by different variables depending on the subgroups of patients studied. Despite the small number of studies carried out on the subject, we were able to identify two relevant factors in the study of the impact of cancer on self-esteem.

First, physical damage related to cancer treatment is common and leads to changes in body image (Batchelor, 2001; Fobair et al., 2006; Paterson et al., 2016; Rzeszutek et al., 2019). This impact on body image would lead to a modification of self-views and could induce a decrease in self-esteem (Pintado, 2017). For example, the level of self-esteem of breast cancer patients is significantly lower in women who received a mastectomy than in women who were able to have breast-conserving surgery (Al-ghazal et al., 2000). Münstedt et al. (1997) showed a significant drop in the self-esteem level of breast cancer patients during their chemotherapy treatment. This decrease in self-esteem persists beyond the regrowth of the patients’ hair. If treatment-induced alopecia can directly impact physical self-perceptions, the authors interpret this prolonged decline in self-esteem by the difficulty for patients to put in place effective coping strategies allowing the maintenance of a positive self-image during and after this type of invasive treatment.

Second, the impact of cancer on self-esteem is thought to be greater in younger adults, as has been shown in women with breast cancer (Penman et al., 1987; Manos et al., 2005) or in patients undergoing chemotherapy (Vidthya et al., 2019). Many age-related variables are also relevant, such as patients’ socioeconomic status (e.g., marital status, employment status). In addition, some cancers (e.g., breast cancer, prostate cancer) and treatments (e.g., surgery, chemotherapy) will have significant side effects and sometimes long-term consequences such as fertility problems or sexual dysfunction (Fobair et al., 2006; Gómez-Campelo et al., 2014), thus impacting the sense of virility/femininity and family plans of some young patients. For the moment, there are still few studies that have focused on the association between age and patients’ self-esteem (Curbow and Somerfield, 1991) and its possible other moderators.

## Discussion

The psychological impact of cancer is significant, with depressive symptoms frequently observed in cancer patients. Self-esteem is a fundamental psychological resource that is highly associated with health behaviors, psychological well-being, and mental health. Given its association with coping and social support, we argue that the preservation of self-esteem appears to be an important clinical issue in oncology care to prevent depression.

However, studies on the effects of self-esteem on the development of depression in cancer patients are scarce, and, surprisingly, the results do not allow to conclude a specific impact of self-esteem in the association between cancer and depression. Indeed, the results on the role of self-esteem in the development of depression in cancer patients facing a stressful life event are mixed. Few studies have examined the effect of self-esteem on the development of depression in patients specifically with cancer. One exception is the study by Heidrich et al. (1994) which investigated the impact of perceived physical health (physical symptoms, level of dependence, and perceived chronicity of cancer) on different psychological adjustment variables in 110 cancer patients. The results of this study showed that the effect of perceived physical health on depressive symptoms was partially mediated by self-discrepancy (difference between the perceived Self and the ideal Self). In another study, Dentale et al. (2018) showed a moderation of the link between stressful life events and depression by the participants’ global level of self-esteem in a longitudinal study of 95 students. However, using data from three longitudinal studies conducted on healthy participants, Orth et al. (2009) were unable to demonstrate the mediating effect of self-esteem on the link between stressful life events and depressive symptoms. These authors conclude here that self-esteem and stressful life events are two independent risk factors for depression. The difficulty in demonstrating the effect of self-esteem on the impact of stressful life events on the development of depression could be explained by Thoits (1995) postulate that psychological resources, such as self-esteem, have an impact on individuals’ psychological well-being independently of the presence of a threatening life event (e.g., cancer). Indeed, the predictive effect of low self-esteem on the development of depressive symptoms has been demonstrated in different types of populations, whether in cancer patients (Schroevers et al., 2003b) or healthy subjects (Sowislo and Orth, 2013) and does not appear to be particularly related to the occurrence of a negative or stressful life event such as cancer.

In this article, we provide an overview of existing research on self-esteem’s involvement in psychological adjustment to cancer. This is an important issue because it has been poorly addressed in the literature and sometimes misunderstood. However, the small number of studies carried out and the heterogeneity of cancer patients limited our ability to draw unambiguous conclusion. It seems important to further investigate the extent to which self-esteem is impacted in subtypes of cancer patients with varying medical conditions (e.g., type of cancer, stage, and location) and socio-demographic status (e.g., age, gender). Despite this need for additional investigations, it remains important to consider self-esteem in cancer patients for several reasons. First, despite the lack of demonstration of a specific link between cancer, self-esteem, and depression, positive self-esteem may reduce the risk of developing depression (in cancer patients or other populations). But this would be even more important for cancer patients since some studies have shown that depression can impact the immune system (Reiche et al., 2004, 2005). In addition, special attention should be paid to specific groups of patients, such as young adult patients or those who experience significant physical damages, in whom cancer may cause a decrease in self-esteem. We think that the early identification of patients most at risk of a decrease in self-esteem and the use of interventions targeting self-perceptions could have preventive effects in these patients by promoting psychological adjustment to cancer. It would allow patients to better adjust with the diagnosis and treatment of cancer, adopting adaptive coping strategies, and avoiding the deterioration of social support, which are also important psychological resources for patients. Taken together, this would allow to decrease the psychological impact of cancer by reducing the risk of developing depressive disorders and would also have positive consequences on patients’ health.

## Data Availability Statement

The original contributions presented in the study are included in the article/supplementary material, further inquiries can be directed to the corresponding author.

## Author Contributions

NN wrote the manuscript. BN and MB supervised and reviewed the manuscript. All authors contributed to the article and approved the submitted version.

## Funding

This work was supported by the “Ligue Contre le Cancer,” Paris, France [3-year doctoral grant], the LPNC, and the LIP/PC2S.

## Conflict of Interest

The authors declare that the research was conducted in the absence of any commercial or financial relationships that could be construed as a potential conflict of interest.

## Publisher’s Note

All claims expressed in this article are solely those of the authors and do not necessarily represent those of their affiliated organizations, or those of the publisher, the editors and the reviewers. Any product that may be evaluated in this article, or claim that may be made by its manufacturer, is not guaranteed or endorsed by the publisher.
